# Inhibition of HSP90 in Driver Oncogene-Defined Lung Adenocarcinoma Cell Lines: Key Proteins Underpinning Therapeutic Efficacy

**DOI:** 10.3390/ijms241813830

**Published:** 2023-09-07

**Authors:** Ángela Marrugal, Irene Ferrer, Álvaro Quintanal-Villalonga, Laura Ojeda, María Dolores Pastor, Ricardo García-Luján, Amancio Carnero, Luis Paz-Ares, Sonia Molina-Pinelo

**Affiliations:** 1 H12O-CNIO Lung Cancer Clinical Research Unit, Instituto de Investigación Hospital 12 de Octubre & Centro Nacional de Investigaciones Oncológicas (CNIO), 28029 Madrid, Spainlpazaresr@seom.org (L.P.-A.); 2 CIBERONC, Instituto de Salud Carlos III, 28029 Madrid, Spain; acarnero@us.es; 3 Program in Molecular Pharmacology, Memorial Sloan Kettering Cancer Center, New York, NY 10065, USA; 4 Instituto de Biomedicina de Sevilla (IBiS) (HUVR, CSIC, Universidad de Sevilla), 41013 Sevilla, Spain; 5 Respiratory Department, Hospital Universitario Doce de Octubre, 28041 Madrid, Spain; 6 Medical Oncology Department, Hospital Universitario Doce de Octubre, 28041 Madrid, Spain; 7 Medical School, Universidad Complutense, 28040 Madrid, Spain

**Keywords:** lung adenocarcinoma, HSP90, HSP90 inhibitors, response biomarkers

## Abstract

The use of 90 kDa heat shock protein (HSP90) inhibition as a therapy in lung adenocarcinoma remains limited due to moderate drug efficacy, the emergence of drug resistance, and early tumor recurrence. The main objective of this research is to maximize treatment efficacy in lung adenocarcinoma by identifying key proteins underlying HSP90 inhibition according to molecular background, and to search for potential biomarkers of response to this therapeutic strategy. Inhibition of the HSP90 chaperone was evaluated in different lung adenocarcinoma cell lines representing the most relevant molecular alterations (EGFR mutations, KRAS mutations, or EML4-ALK translocation) and wild-type genes found in each tumor subtype. The proteomic technique iTRAQ was used to identify proteomic profiles and determine which biological pathways are involved in the response to HSP90 inhibition in lung adenocarcinoma. We corroborated the greater efficacy of HSP90 inhibition in EGFR mutated or EML4-ALK translocated cell lines. We identified proteins specifically and significantly deregulated after HSP90 inhibition for each molecular alteration. Two proteins, ADI1 and RRP1, showed independently deregulated molecular patterns. Functional annotation of the altered proteins suggested that apoptosis was the only pathway affected by HSP90 inhibition across all molecular subgroups. The expression of ADI1 and RRP1 could be used to monitor the correct inhibition of HSP90 in lung adenocarcinoma. In addition, proteins such as ASS1, ITCH, or UBE2L3 involved in pathways related to the inhibition of a particular molecular background could be used as potential response biomarkers, thereby improving the efficacy of this therapeutic approach to combat lung adenocarcinoma.

## 1. Introduction

Cancer is one of the leading health concerns in the world and is expected to cause approximately 11.5 million deaths by 2030 [1]. Among the different types of tumors, lung cancer is of particular note as it is responsible for one in five cancer deaths and consequently represents the highest mortality rate worldwide [2,3]. Histologically, lung cancer can be classified into non-small cell lung cancer (NSCLC) and small cell lung cancer (SCLC), accounting for 85% and 15% of cases, respectively. The heterogeneity present in NSCLC has led to the sub-classifications of adeno-carcinoma (50%), squamous cell carcinoma (35%), and large cell carcinoma (15%) being applied [4]. In addition to histological variability, different genetic alterations underlie each subtype, opening up a range of possibilities in the development of therapies aimed at specific alterations [5,6,7]. Specifically, in the most common histological subtype, lung adenocarcinoma, several genomic alterations in driver genes have been identified, including epidermal growth factor receptor (EGFR), echinoderm microtubule-associated protein-like protein 4 fused to anaplastic lymphoma kinase (EML4-ALK), Kirsten rat sarcoma viral oncogene homolog (KRAS), serine/threonine-protein kinase B-Raf (BRAF), mesenchymal-epithelial transition (MET) factor, human epidermal growth factor 2 (HER2/ErbB2/neu), or ROS pro-to-oncogene 1, receptor tyrosine kinase (ROS1) [8,9]. This has permitted the development of therapies targeting specific driver oncogenes in lung adenocarcinoma [10,11,12]. However, despite advances in precision medicine, many cases of lung adenocarcinoma still lack an effective targeted therapy [13], and in those cases where a therapy exist, the development of acquired resistance is problematic [14,15,16].

Based on the above, a broad-spectrum therapy directed simultaneously against several driver oncogenes would be of significant benefit. In this regard, the 90 kDa heat shock protein (HSP90) stands as a therapeutic target due to its ability to regulate and stabilize a large number of oncogenic proteins, or so-called HSP90 clients. The main isoforms of this protein, inducible HPS90α and constitutive HSP90β, act as molecular chaperones of the client proteins by promoting their folding and maturation, as well as regulating their stability, activity, and function [17,18]. In this way, HSP90 inhibition has been shown to result in a rapid de-crease of client protein activity and subsequent degradation, simultaneously de-creasing multiple oncoproteins and thus modulating features of the malignant phenotype [19,20]. Currently, the most advanced inhibitors are those that block HSP90′s ATPase activity, which is essential for its function as a molecular chaperone, thereby leading to the proteasomal degradation of client proteins [19,21,22]. This fact is particularly relevant in lung adenocarcinoma, since some of the oncodrivers of this histological subtype are HSP90 clients such as EGFR [23], HER2 [24], MET [25], BRAF [26], and the EML4-ALK fusion protein [27]. The degradation of these driver proteins after HSP90 inhibition leads to loss of tumor cell viability [28], while decreased expression of the HSP90 gene is associated with increased survival of NSCLC patients [29]. On the other hand, the elevated expression of this chaperone has been associated with resistance to chemotherapy and radiotherapy [30,31].

Taken together, these data support the notion of HSP90 inhibition as a therapeutic strategy and point to the significant potential for clinical trials in NSCLC to be per-formed [32]. Some of the most encouraging HSP90 inhibitors identified to date have been the geldanamycin derivatives tanespimycin (17-AAG) and retaspimycin hydrochloride (IPI-504), as well as the radicicol derivatives ganetespib (STA-9090) and luminespib (AUY-922) [33]. These have been responsible for some of the most promising results in clinical trials, especially those in which the stratification of NSCLC was carried out based on the presence of molecular alterations [34,35,36]. This is mainly due to the fact that some driver proteins, such as EGFR or EML4-ALK exhibit a strong dependence on HSP90 that makes them more sensitive to HSP90 inhibition. Consequently, this translates into an increased efficacy of HSP90 inhibition in patients with tumors that are ‘molecularly addicted’ to these proteins [37]. However, not all patients whose tumors are sensitive to HSP90 inhibitors respond positively to treatment, meaning that the use of HSP90 inhibition as a therapy in lung adenocarcinoma remains limited due to moderate drug efficacy, the emergence of drug resistance, and early tumor recurrence [38]. Advances in this field have so far focused on expanding our understanding of the biological basis of HSP90 inhibition in NSCLC [39], as well as identifying biomarkers that predict response to inhibitors of these tumors [40]. In addition, to improve the efficacy of HPS90 inhibitors, it is essential to monitor inhibition responses to ensure proper blocking of the chaperone. To date the clinical trial studies of pharmacodynamic effects after HSP90 inhibition have been based primarily on the analysis of HSP70 induction, although a significant correlation between this biomarker and treatment response has not yet been validated [41].

For these reasons, the identification of additional predictors to optimize the clinical efficacy of HSP90 inhibitors in lung adenocarcinoma is essential and forms the main objective of the research outcomes reported here. For this purpose, isobaric tags for relative and absolute quantification (iTRAQ), a high-throughput proteomics technique widely used in NSCLC research [42,43,44], have been selected. This proteomic assay was used to identify proteins related to HSP90 inhibition responsiveness according to the most relevant molecular alteration in lung adenocarcinoma such as EGFR and KRAS mutations and ALK translocation. This work aims to identify proteins that could serve as potential biomarkers to monitor response to HSP90 inhibitors, thereby improving the efficacy of this therapeutic strategy in lung adenocarcinoma.

## 2. Results

### 2.1. Expression of HSP90 and Related Proteins in Different Molecular Subtypes of Lung Adenocarcinoma

**Figure 1:**
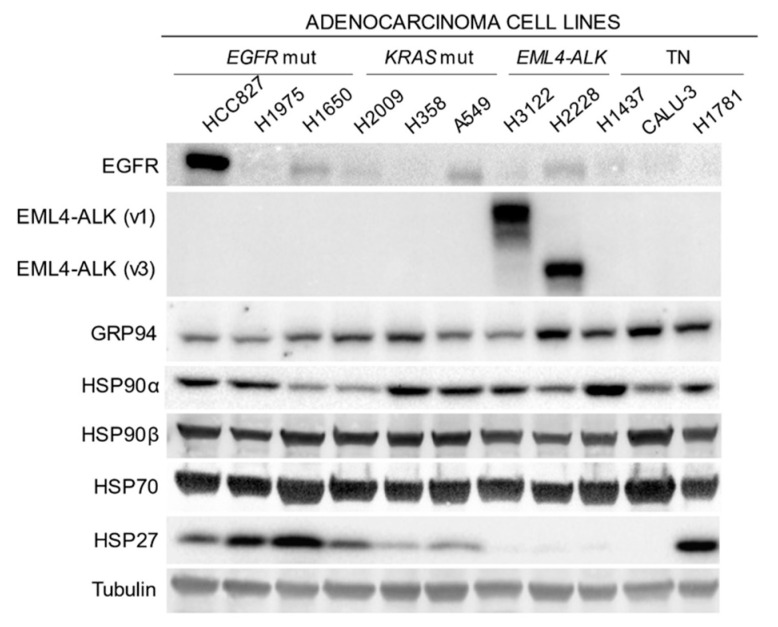
Characterization of the lung adenocarcinoma cell lines panel used. Study of HSP90 protein expression, other related heat shock proteins, and EGFR and EML4-ALK client proteins in the cell lines under study. EGFR = EGFR mutation, KRAS = KRAS mutation, ALK = EML4-ALK translocation carrier, TN = triple negative (EGFR, KRAS and wild-type ALK).

In the analyzed panel of 11 lung adenocarcinoma cell lines with distinct molecular patterns, the expression of HSP90α, HSP90β, and other related HSPs was determined (Figure 1).

HSPP90α expression was different in each molecular sub-group, with the lowest levels found in H1650 for the EGFR-mutated subtype, H2009 for the KRAS-mutated subtype, H2228 for the EML4-ALK -translocated subtype, and CALU-3 among the Triple Negative (TN) cell lines (referring to the absence of alterations in EGFR, KRAS, and ALK). No significant changes in HSP90β or HSP70 expression were found in the different molecular groups studied. However, it is noteworthy that within each molecular subtype, the cell lines with the lowest expression of HSP90α showed a higher expression of HSP90β and HSP70, with the exception of the EML4-ALK subtype. Similar to GRP94 (heat shock protein 90 beta family member 1) expression, generally showed a reverse expression pattern to HSP90α. Finally, it should be noted that HSP27 was the only chaperone that showed differential expression according to the molecular sub-group, with the highest expression levels seen in EGFR-mutated cell lines, inter-mediate levels in the KRAS-mutated cell lines, and undetectable expression in the EML4-ALK-translocated lines. The exception was the TN group where large variations were observed.

Expression levels of the most interesting HSP90 client proteins were also studied. Focusing on EGFR, the highest expression of this mutated receptor was detected in the HCC827 cell line. The H1650, H2009, A549, and H2228 cell lines, each of which has a different mutational status of this receptor, presented high levels of EGFR expression, while the remaining cell lines presented low or undetectable expression of this receptor. It, therefore, follows that the EGFR mutation status is not related to EGFR expression. In contrast, the EML4-ALK fusion protein was detected exclusively in cell lines harboring variant 1 (H3122) and variant 3 (H2228) of this translocation.

### 2.2. Effect of Inhibition of HSP90 According to the Lung Adenocarcinoma Molecular Subgroup

IC80 (80% inhibitory concentration) values of selected HSP90 inhibitors were determined to assess the sensitivity of the lung adenocarcinoma cell lines (Table 1). Lower IC80 values were seen in most cell lines in response to treatment with radicicol derivatives (STA-9090 and AUY-922), whereas higher values were detected after treatment with geldanamycin derivatives (17-AAG and IPI-504). Focusing on the molecular subgroups, EGFR-mutated cell lines were the most sensitive to all HSP90 inhibitors, followed by cell lines with EML4-ALK translocation. In contrast, the triple-negative cell lines, particularly CALU-3, were the most resistant to both inhibitor families.

**Figure 2:**
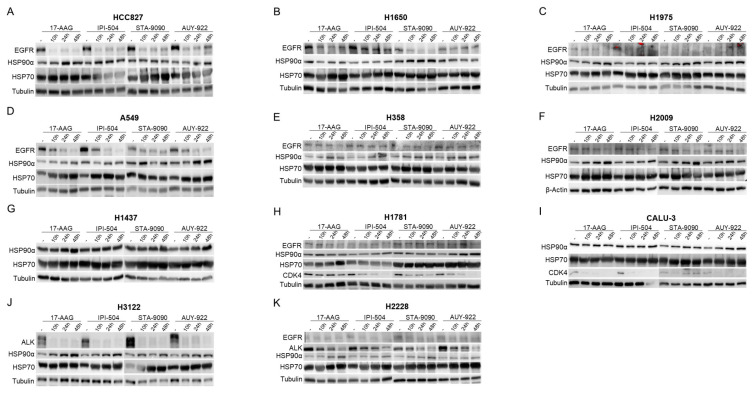
Evaluation of HSP90 inhibition in lung adenocarcinoma cell lines. (**A**) HCC827, (**B**) H1650, (**C**) H1975, (**D**) A549, (**E**) H358, (**F**) H2009, (**G**) H1437, (**H**) H1781, (**I**) CALU-3, (**J**) H3122 and (**K**) H2228 were subjected to IC80 concentration of 17-AAG, IPI-504, STA-9090 and AUY-922 for 10, 24 or 48 h before Western blot analysis to study expression of HSP90α, HSP70 and the corresponding EGFR, EML4-ALK or CDK4 client proteins. Each experiment was performed in triplicate. Western blots correspond to a representative image of the replicates. - = untreated with inhibitor; h = hours.

Protein expression responses of the cell line panel were evaluated after treatment with the different HPS90 inhibitors at their IC80 concentration (Figure 2). A compensatory induction of HSP70 and HSP90 expression was identified by Western blot analysis, confirming the correct inhibition of HSP90. We also found that the effect of HSP90 inhibition on client proteins depended on the molecular subgroup of the cell line. The degradation of EGFR was more pronounced and faster in EGFR-mutated cell lines such as HCC827 (Figure 2A) and H1650 (Figure 2B). On the other hand, in cell lines where EGFR was not the oncogenic driver, their EGFR degradation was much lower even though it was highly expressed in the cell line, as was the case in the A549 (Figure 2D), H358 (Figure 2E), H2009 (Figure 2F), H1781 (Figure 2H) and H2228 (Figure 2K) cell lines.

EML4-ALK was degraded after HSP90 inhibition in the two cell lines showing expression of this fusion protein. However, in the H3122 line that carries translocation variant 1, the protein was completely degraded in response to all inhibitors from the beginning of treatment (Figure 2J). H2228, which harbors translocation variant 3, showed less degradation, especially after treatment with geldanamycin derivatives (Figure 2K).

### 2.3. HSP90 Gene Silencing in Lung Adenocarcinoma Cell Lines

**Figure 3:**
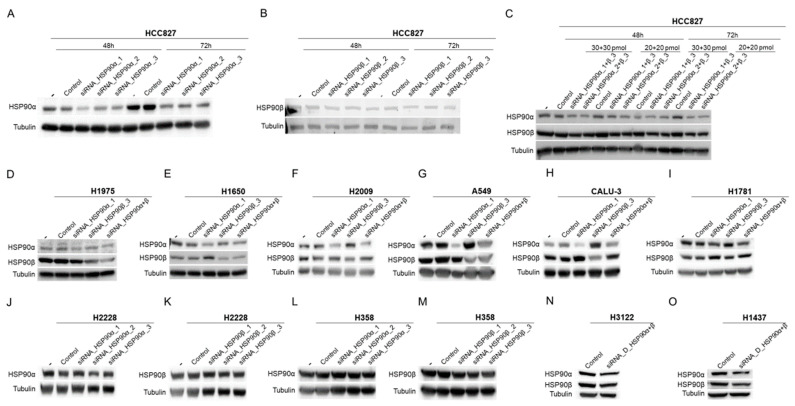
Effectiveness of HSP90 gene silencing in the panel of lung adenocarcinoma cell lines. Western blot analysis of the validity of gene silencing of (**A**) HSP90α and (**B**) HSP90β in the HCC827 cell line using siRNAs from the commercial company Origene. In this same cell line, we chose a 48-h incubation with 30 pmol of siRNA_HSP90α_1 and siRNA_HSP90β_3 as the optimal conditions for (**C**) combined silencing of HSP90. HSP90α and HSP90β expression for the established conditions was studied in the (**D**) H1975, (**E**) H1650, (**F**) H2009, (**G**) A549, (**H**) Calu-3, and (**I**) H1781 cell lines. Verification of conditions for optimal silencing of HSP90α and HSP90β in (**J**,**K**) H2228 and (**L**,**M**) H358. Verification of HSP90α and HSP90β protein reduction in response to treatment with siRNAs (Dharmacon) of the (**N**) H3122 and (**O**) H1437 cell lines. - = Untransfected; Control = non-specific interfering RNA; siRNA_HSP90α = interfering RNA against HSP90α; siRNA_HSP90β = interfering RNA against HSP90β.

To define specific responses to HSP90 inhibition and to detect off-target effects of pharmacological inhibitors of HSP90, the expression of both isoforms of this chaperone was silenced in cell lines (Figure 3). Due to the relevance of HSP90 in cell viability, halving the expression of this chaperone was considered suitable as a positive control of inhibition.

Silencing conditions were selected in the HCC827 line by assessing the decrease in expression of HPS90α (Figure 3A), HSP90β (Figure 3B), and the combination of both (Figure 3C) under different conditions. Treatment with the most effective interfering RNAs (siRNA_HSP90α_1 and siRNA_HSP90β_3) at 30 pmol and for 48 h achieved an optimum reduction in expression and was therefore used in EGFR-mutated H1975 (Figure 3D), H1650 (Figure 3E), KRAS-mutated H2009 (Figure 3F), A549 (Figure 3G), triple-negative CALU-3 (Figure 3H), and H17881(Figure 3I) cell lines. On the other hand, in the H2228 (EML4-ALK-trasnslocated) and H358 (KRAS-mutated) cell lines, enhanced silencing of HSP90α was observed with siRNA_HSP90α_2 (Figure 3J) and siR-NA_HSP90α_3 (Figure 3L), while siRNA_HSP90β_3 was also used for HSP90β silencing (Figure 3K,M). Finally, in the H3122 (EML4-ALK translocation bearer) (Figure 3N) and H1437 (TN) (Figure 3O) cell lines, other siRNAs (Dharmacon, Lafayette, CO, USA) had to be used to silence HSP90.

### 2.4. Differentially Expressed Proteins Underlying the HSP90 Inhibition Identified by Proteomic Profiling

**Figure 4:**
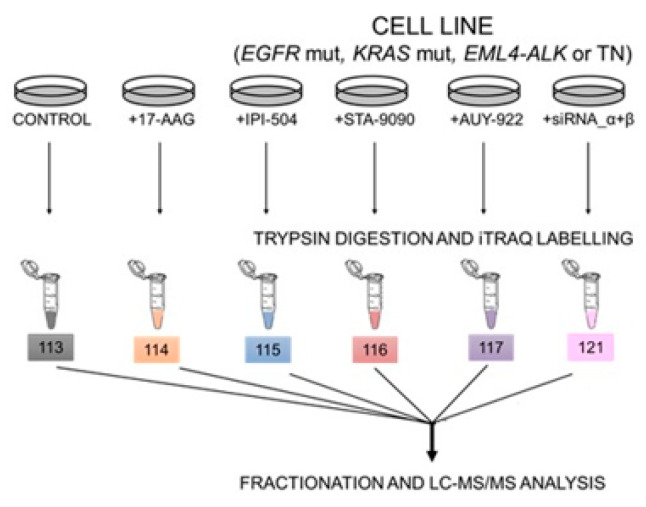
iTRAQ design of experiments. 100 μg of proteins from the different conditions to be analyzed, for each cell line under study, were digested with trypsin and labeled with iTRAQ reagents. Each of the thirteen fractions obtained was analyzed by LC-MS/MS and the data were combined to make the corresponding protein identification and quantification. VER = VER-155008; siRNA_α + β = gene silencing of HSP90α and HSP90β.

Isobaric tag for relative and absolute quantitation (iTRAQ) coupled with mass spectrometry (NanoLC-MS/MS) analysis was used to identify those proteins with clinically significant expression changes after inhibition or silencing of HSP90 in each cell line (Figure 4, Table 2 and Supplementary Tables S1–S11).

The CALU-3 and H2228 cell lines had the highest number of proteins showing significant expression changes, while H1650 and H1437 showed the lowest number of proteins that underwent expression changes. These last two cell lines presented a set of values far from the other cell lines and were considered outliers. We found that most cell lines and study conditions resulted in similar expression patterns, with more underexpressed than overexpressed proteins. Specifically, in the H1975, H2228, and H1781 cell lines treated with IPI-504, the number of over-expressed proteins was never higher than that of underexpressed proteins. In contrast, in gene silencing experiments on the H3122 cell line, a higher number of overexpressed proteins was detected.

Considering the total number of proteins with significantly altered expression, six times more deregulated proteins were detected after siRNA treatment than in response to HSP90 inhibitors. Differentiating between underexpressed and overexpressed proteins, the 17-AAG inhibitor was the only treatment in which a higher number of underexpressed proteins was detected, compared to that seen with genetic silencing.

### 2.5. Effect of HSP90 Inhibition on Proteomic Profiling with Respect to the Lung Adenocarcinoma Molecular Subgroup

PERSEUS software was used to identify proteins with significant expression changes after HSP90 inhibition in cell lines with the same molecular background (EGFR-mutated, KRAS-mutated, EML4-ALK-translocation, and TN). As shown in Table 3, the highest number of proteins whose expression was significantly altered was found in KRAS-mutated cell lines, while the lowest number was identified in the group of EML4-ALK translocated cell lines.

**Figure 5:**
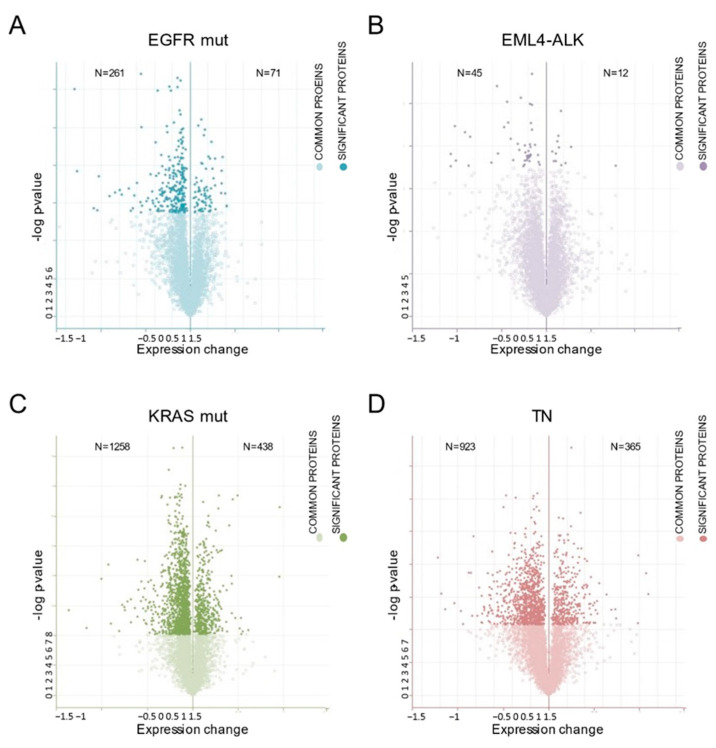
Representation of the significantly altered expression of proteins after HSP90 inhibition in the different lung adenocarcinoma molecular subtypes. Volcano plot of differentially expressed proteins after HSP90 inhibition and their corresponding *p*-value for (**A**) EGFR mutation, (**B**) EML4-ALK translocation, (**C**) KRAS mutation and (**D**) triple-negative cell lines. The *X*-axis corresponds to the change in expression of overexpressed (positive values) and underexpressed (negative values) proteins. The *Y*-axis represents the *p*-value of the corresponding change in expression.

Differentially expressed proteins were plotted according to their change in expression and the corresponding *p*-value (Figure 5). All molecular subgroups were identified as having similar expression patterns, with approximately three times more underexpressed than overexpressed proteins after inhibition by HSP90.

Subsequently, proteins whose expression was significantly altered and which were also deregulated in gene silencing were identified to ensure the real effect of inhibition (Table 3). Based on this selection, the final number of proteins decreased by approximately 30% in all lung adenocarcinoma molecular subgroups.

**Figure 6:**
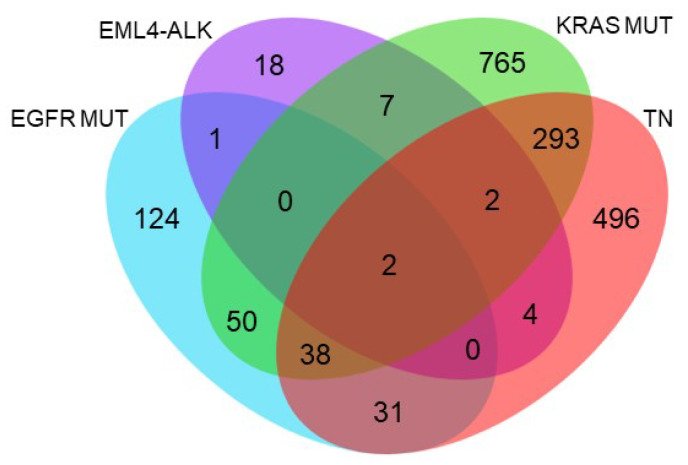
Venn diagram showing proteins that were significantly and specifically deregulated after HSP90 inhibition in the different lung adenocarcinoma molecular subgroups. EGFR mut = group of cell lines with EGFR mutation, EML4-ALK = group of cell lines with translocation in ALK, KRAS mut = group of cell lines with KRAS mutation, TN = group of triple-negative cell lines.

A Venn diagram was then used to represent those proteins specific to HSP90 inhibition that were significantly deregulated for each molecular subtype (Figure 6). Only two proteins were deregulated after inhibition of HSP90 inhibition in all molecular subgroups studied. In particular, the overexpression of acireductone dioxygenase 1 (ADI1) and underexpression of ribosomal RNA processing protein 1 (RRP1) were identified. Moreover, approximately half of the proteins showing significantly altered expression were specific to molecular subgroups. Consequently, the number of proteins among the remaining combinations was also low, especially when one of the molecular subtypes in the comparison was the translocated EML4-ALK.

### 2.6. Functional Annotation of Specifically and Significantly Down-Regulated Proteins after HSP90 Inhibition in Different Molecular Subgroups of Lung Adenocarcinoma

Table 3 shows the biological pathways associated with proteins that were specifically and significantly deregulated after HSP90 inhibition. The highest number of biological pathways was identified in the KRAS-mutated cell lines, which was to be expected since this molecular subtype had the highest number of differentially expressed proteins. However, it should be noted that in the EML4-ALK cell lines, a similar number of biological pathways was detected as in the TN cell lines, and a higher number than in the EGFR-mutated cell lines, even though these molecular groups had 25 and 7 times more deregulated proteins, respectively. The small group of deregulated proteins after HSP90 inhibition in translocated EML4-ALK cell lines suggests that a large number of biological pathways are involved in such inhibition (Table 4).

**Figure 7:**
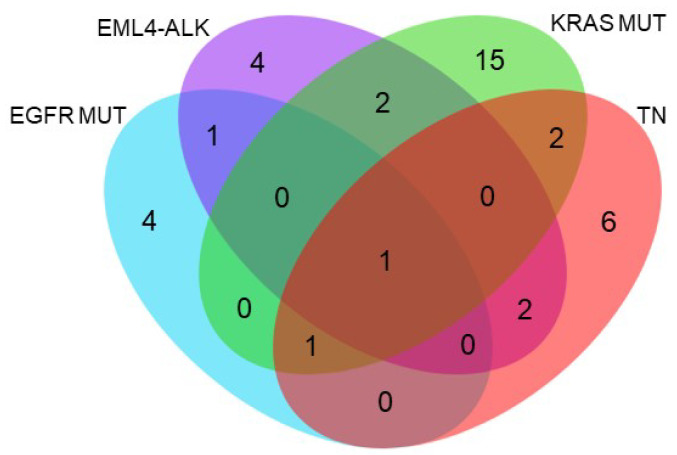
Classification of altered pathways after HSP90 inhibition in the different lung adenocarcinoma molecular subgroups. EGFR mut = group of cell lines with mutation in EGFR, EML4-ALK = group of cell lines with translocation in ALK, KRAS mut = group of cell lines with KRAS mutation, TN = group of triple-negative cell lines.

These enriched biological pathways were classified using a Venn diagram (Figure 7). Apoptosis was the only pathway common to all molecular subgroups, thereby demonstrating the influence of inhibitors on cell death in lung adenocarcinoma. Molecular groups with EGFR mutations or EML4-ALK translocation shared the enrichment of arginine biosynthesis. Alterations in the EGFR and FGF signaling pathways, both of which are related to cell growth, were common to the EML4-ALK translocated and KRAS-mutated molecular subgroups, whereas the EML4-ALK translocated and TN groups shared enrichment of the PDGF and GnRHR signal-ling pathways. Finally, DNA replication and the ubiquitin-proteosome pathway were shared between the KRAS-mutated and TN subtypes. Since proteosome ubiquitination and degradation are essential steps in response to HSP90 inhibition, this alteration could be the result of a differential reaction in the KRAS-mutated and TN groups compared to the other lung adenocarcinoma molecular subgroups.

### 2.7. ADI1 and RRP1 mRNA Expression Are Strongly Associated with Clinical Outcome

**Figure 8:**
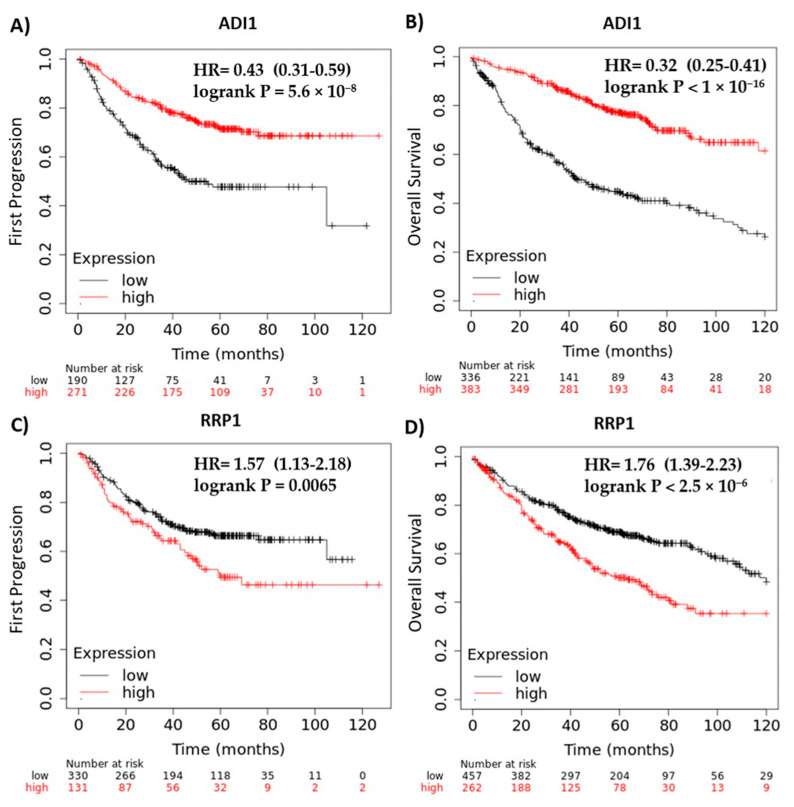
In silico validation of ADI1 and RRP1 as response biomarkers of HSP90 inhibition in lung adenocarcinoma (**A**–**D**). KM plot of ADI expression according to first progression of disease (**A**) and overall survival (**B**), together with relationship of RRP1 expression with first progression (**C**) or overall survival (**D**) of lung adenocarcinoma patients.

To evaluate whether ADI1 y RRP1 are associated with clinical outcomes in patients with lung adenocarcinoma, we analyzed their mRNA expression levels according to disease progression and overall survival by way of the KM Plotter web-site (https://kmplot.com), accessed on 1 July 2022 (Figure 8). An online tool that includes gene expression data and clinical characteristics of 10 independent datasets, published in the Cancer Biomedical Informatics Grid (caBIG), the Gene Expression Omnibus (GEO), and The Cancer Genome Atlas (TCGA) (https://www.cancer.gov/tcga, accessed on 1 July 2022) repositories. We found that lower ADI1 expression was significantly associated with a reduced time to initial disease progression (hazard ratio (HR) = 0.43, 95% confidence interval (CI) = 0.31–0.59, *p* < 0.001) (Figure 8A) and poorer overall survival (HR = 0.32, 95% CI = 0.25–0.41, *p* < 0.001) (Figure 8B). In the case of RRP1, the opposite was true; high mRNA levels were associated with poorer clinical outcomes. Differences were also significant with respect to time to initial disease progression (HR = 1.57, 95% CI = 1.13–2.18, *p* = 0.006) (Figure 8C) and overall survival (HR = 1.76, 95% CI = 1.39–2.23, *p* < 0.001) (Figure 8D).

## 3. Discussion

In the present work, an iTRAQ-based high-throughput quantitative proteomics technique was used to evaluate molecular context-dependent responses to different HSP90 inhibitors and to identify proteins that could potentially be used as biomarkers of the response to inhibition of this chaperone in lung adenocarcinoma. Taken together, the results presented here aim to optimize the use of HSP90 as a therapeutic target in this disease where there are no good response biomarkers available to guide who might benefit from HSP90 inhibitor treatment, or for how long. As a first approach to the study, the efficacy of four HSP90 inhibitors was evaluated on a panel of previously characterized lung adenocarcinoma cell lines. In our experiments, all of the cell lines studied showed compensatory expression of HSP70 after HSP90 inhibition. These results confirm the correct blockade of HSP90 in our study panel, since increased HSP70 expression is thus far the only biomarker used to monitor response to HSP90 inhibition [41]. In addition, our results show that these cell lines were more sensitive to radicicol derivatives (STA-9090 and AUY-922) than to geldanamycin derivatives (17-AAG and IPI-504). Since radicicol derivatives are second-generation inhibitors and have lower off-target toxicities [14], their higher efficacy was expected. Also, our results show how the effectiveness of the inhibitors used was directly related to the oncogenic addiction of the cell line to HSP90. The most significant examples were in the translocated EML4-ALK cell lines, particularly the H3122 carrying variant 1, which showed rapid and complete degradation of the receptor. This may be due to an extremely unstable protein structure that is dependent on HSP90, as described by Richards et al. [45]. As expected, promising results have been obtained in clinical trials of different HSP90 inhibitors in subgroups of patients with translocation of EML4-ALK [32,37].

In relation to these findings, we studied responses to HSP90 inhibition according to the most clinically relevant lung adenocarcinoma molecular subtypes. In our experiments, an overall higher number of underexpressed proteins was detected following HSP90 inhibition given that HSP90 client proteins dissociate from this chaperone after its inhibition and are degraded via the proteosome [22,46].

Subsequently, proteins differentially expressed significantly and specifically after HSP90 inhibition in the different molecular subtypes of lung adenocarcinoma were selected. Of all the proteins identified, ADI1 and RRP1 were consistently deregulated in all the subgroups studied. This low percentage of deregulated proteins suggests that the response to HSP90 inhibition is, at the protein level, highly dependent on the molecular context. Both of these common proteins may be relevant due to their potential use in monitoring response to the HSP90 inhibitors studied here. ADI1 is an acireductone dioxygenase that forms part of the methionine salvage pathway [47]. However, the first identified function of this protein was the binding and inhibition of membrane type 1-matrix metalloproteinase (MT1-MMP), an oncogenic protein involved in tumor invasion and progression [48,49,50]. Based on this evidence, and due to the reduced expression of ADI1 in different tumor types, this protein has been proposed as a possible tumor sup-pressor in several types of cancer [48,49,51,52]. Among the mechanisms by which this protein could function as a tumor suppressor is the correlation of ADI1 overexpression with a higher rate of apoptosis, which could be a consequence of the increase in metabolites produced by this enzyme in the methionine salvage pathway [51,52]. Furthermore, in our study, a higher expression of ADI1 was shown to be correlated with better disease progression outcomes and overall survival in patients with lung cancer. Therefore, an increased expression of ADI1, detected after inhibition of HSP90 inhibition in all molecular subtypes of lung adenocarcinoma, proved to be a potential indicator of adequate response.

On the other hand, RRP1 was described as a key factor in ribosome biogenesis [53]. To date, the involvement of RRP1 has been demonstrated both in the cleavage of the 47S ribosomal RNA precursor transcript [54], and in the physical separation of both precursors [55] that gives rise to the small and large subunits of the ribosome. Since both processes are essential for ribosome biogenesis in human cells, the lower expression of RRP1 detected in our experiments threatens protein synthesis and directly affects cell viability. Consistent with this, lower RRP1 expression correlates with improved disease progression outcomes and overall survival in lung cancer. Therefore, the expression of this protein could be considered a good predictive biomarker of the antitumoral effectiveness of HSP90 inhibitors, regardless of the molecular subtype of lung adenocarcinoma.

From another point of view, and based on the results obtained through functional annotation, we highlight that the cellular apoptosis pathway was found to be altered in all molecular subgroups studied after HSP90 inhibition. These results agree with those previously obtained by two-dimensional gel electrophoresis where, after treatment of different cell lines with HSP90 inhibitors, one of the common pathways was apoptosis [39]. On this basis, and since low HSP90 activity induces apoptosis in lung cancer [56,57], our results suggest that this cell death pathway is a key process during the pharmacological inhibition of HSP90 in lung adenocarcinoma, regardless of the molecular subtype and inhibitor used.

On the other hand, dysregulation of the arginine biosynthesis pathway was identi-fied in cell lines showing EGFR mutations and EML4-ALK translocation. Arginine is a semi-essential amino acid that becomes essential in tumor growth, mainly due to the high energy demand required to maintain intense proliferation [58]. Argininosuccinate synthase 1 (ASS1), an arginine-metabolizing enzyme, is overexpressed in several tumor types, such as lung, colon, gastric, and ovarian cancers [59]. It is possible that high levels of ASS1 support tumor proliferation and aggressiveness through increased arginine, which translates into increased nitric oxide (NO) production [60]. High concentrations of NO have been reported to cause a cytotoxic effect in the cell due to the induction of DNA damage, as well as gene mutations followed by apoptosis [61]. Therefore, elevating NO levels through donor drugs has been used as a therapeutic strategy to reduce tumor progression and increase tumor blood flow, which enhances the delivery of cytotoxic therapy to tumor tissue [62,63]. Specifically, the potential of the NO donor glyceryltrinitrate as a chemo-sensitizing agent was demonstrated in NSCLC [64], while pre-treatment with the NO donor RRx-001 results in the sensitization of carboplatin-refractory patients [65]. These results agree with those obtained in our cell lines characterized by EML4-ALK translocation where, after HSP90 inhibition, there was an increase in ASS1. This phenomenon could induce cytotoxicity via an excess of NO in this molecular subtype, as well as enable a possible therapeutic combination of drugs to be used. In addition, in the exclusively EGFR-mutated cell lines, an underexpression of ASS1 was detected after treatment with HSP90 inhibitors. Different tumor types show variability in the expression of this enzyme, which makes cancer cells dependent on or independent of exogenous arginine [66]. In the case of dependence, arginine deprivation has been confirmed to be an excel-lent therapeutic strategy [67,68]. However, these tumors often develop resistance to deprivation-inducing agents using cytosolic aspartate, which is not used by ASS1, or by the CAD enzyme complex for pyrimidine nucleotide synthesis and cell proliferation [69]. However, in EGFR-mutated cell lines, we detected, in addition to ASS1 underexpression, a reduction in CAD after HSP90 inhibition. This double protein decrease could prevent or at least weaken the previously described proliferative mechanism of ASS1-deficient cells. Taken together, these results support the deregulation of arginine synthesis after HSP90 inhibition as a key mechanism with the potential to block cell proliferation in lung adenocarcinoma with mutated EGFR or EML4-ALK translocation. This concept requires further study.

The ubiquitin-proteosome and DNA replication pathways were altered in KRAS-mutated and triple-negative molecular subtypes of lung adenocarcinoma after HSP90 inhibition. Since ubiquitination and degradation in the proteosome are essential steps in the response to HSP90 inhibition [22,46], the differential expression of two essential proteins in this pathway and in both molecular sub-types are highlighted here: ITCH (E3 ubiquitin-protein ligase itchy homolog) and UBE2L3 (ubiquitin-conjugating enzyme E2 L3). ITCH indirectly inhibits the Wnt/β-catenin signaling pathway [70], whose aberrant activation is crucial for the initiation, progression, and metastasis of lung cancer [71,72]. Therefore, increased expression of ITCH, which is normally underexpressed in lung cancer, regulates cell proliferation by blocking the Wnt/β-catenin pathway [73]. In our study, this protein was found to be underexpressed in the KRAS-mutated and triple-negative subgroups, while it was overexpressed under most conditions in the EGFR-mutated and EML4-ALK translocation molecular subtypes. Therefore, we suggest that this E3 ubiquitin ligase could be involved in the differential responses seen after HSP90 inhibition, being more effective in molecular subgroups (i.e., EGFR mutation and EML4-ALK translocation) where ITCH expression was in-creased. UBE2L3 has been linked to the stability of the tumor suppressor p53-binding protein 1, c-FOS, or the NF-κB precursor p105, demonstrating the relationship between this enzyme and carcinogenesis [74,75]. Consequently, overexpressed UBE2L3 was detected in different tissues and cell lines of NSCLC, and its expression level has been directly related to the cancer stage in patients. Based on the above, UBE2L3 was proposed as a potential therapeutic target in NSCLC [76]. In our results, this enzyme was found to be overexpressed in the triple-negative and KRAS-mutated molecular subgroups, where a clear dysregulation of the ubiquitin-proteosome pathway was detected. In contrast, this enzyme was found to be significantly underexpressed in molecular subtypes with EGFR mutations and EML4-ALK translocation, which could be related to a more effective response of these cell lines to HSP90 inhibition. In general, these data indicate that UBE2L3 overexpression and/or underexpression of ITCH could favor cell proliferation in the KRAS-mutated and triple-negative lung adenocarcinoma subgroups, hindering the antitumor potential of HSP90 inhibitors in these contexts.

Based on the above and taking into account the limitations of the study performed, which was based exclusively on in vitro data, ongoing study and in vivo validation of potential biomarkers and the mechanisms of response to HSP90 inhibition in lung adenocarcinoma proposed here are required to substantiate their importance. Such limitations are due to a lack of samples from patients treated with HSP90 inhibitors, mainly as a consequence of the toxicity profiles of clinically tested inhibitors and the paucity of response to treatment by patients with lung adenocarcinoma. However, the combined treatment with HSP90 inhibitors along with activating or blocking agents of proteins or biological pathways proposed as relevant in the response could decrease the pharmacological dose required and thus the toxicity to which patients are exposed. Furthermore, the identification of molecular con-text-dependent proteins proposed as potential biomarkers of response would facilitate monitoring outcomes in patients with lung adenocarcinoma following treatment with HSP90 inhibitors.

## 4. Materials and Methods

### 4.1. Cell Line Culture

Eleven human lung adenocarcinoma cell lines (EGFR-mutated: HCC827, H1650, and H1975; KRAS-mutated: A549, H2009, and H358; ALK translocation bearer: H3122 and H2228; and Triple Negative (TN) referring to the absence of alterations in EGFR, KRAS, and ALK: CALU3, H1437, and H1781) were used for this study. All cell lines were obtained from the American Type Culture Collection (ATCC), with the exception of the H3122 cell line, which was kindly provided by Dr. Koivunen. Cells were cultured in RPMI-1640 medium (Sigma-Aldrich, St. Louis, MO, USA), with the exception of the A549 cell line, which was propagated in DMEM medium (Sigma-Aldrich, St. Louis, MO, USA). Both media were supplemented with 10% fetal bovine serum (FBS, TICO Europe), 1% antibiotic-antimycotic solution (Sigma-Aldrich, St. Louis, MO, USA), and 1% glutamine (*v*/*v*). All cell lines were cultured as monolayers at 37 °C and 5% CO_2_ in a humidified incubator. Cells were authenticated and periodically checked to ensure the absence of mycoplasm.

### 4.2. Genetic Silencing of HSP90

Cells were seeded at appropriate densities into 35 mm culture dishes and incubated at 37 °C and 5% CO_2_ to reach 70% confluence 24 h later. HSP90 small interfering RNAs (siRNAs) were transiently transfected into cells with LipofectamineTM RNAiMAX (Invitrogen, Waltham, MA, USA), according to the manufacturer’s instructions. At least two of four different siRNAs (SR302262 and SR302264) from Origene were used to induce silencing of HSP90α and HSP90β, respectively. Furthermore, cells were transfected with scramble siRNA (SR30002, Origene) as a negative control. Finally, simultaneous transfections were performed in each cell line with the aim of silencing both genes at the same time. Transfected cells were cultured for 48–72 h before being used for further analyses.

### 4.3. Treatment with HSP90 Inhibitors

For HSP90 inhibition studies, derivatives of geldanamycin (tanespimycin (17-AAG) (Selleckchem, Munich, Germany) and retaspimycin hydrochloride (IPI-504) (Eurodiagnóstico, Madrid, Spain) and derivatives of radicicol (ganetespib (STA-9090) and luminespib (AUY-922) from Selleckchem, Munich, Germany) were used. All HSP90 inhibitors were dissolved in dimethyl sulfoxide (DMSO) according to the manufacturer’s instructions for in vitro application.

Cell line drug sensitivity was measured using fluorescence-based cell viability assays after 96 h of treatment with the different HSP90 inhibitors at concentrations ranging from 0.33 nM to 20 uM. Three independent experiments were performed at each concentration. Dose-response curves made it possible to calculate half-maximal inhibitory concentration values (IC50). Following this, the concentration of each HSP90 inhibitor at which growth was reduced to 80% (IC80) was calculated and applied to cell lines in the log phase seeded at 3 × 10^3^ cells/well in 96-well plates for 24 h.

### 4.4. Western Blot (WB)

Total protein extracts from each treated cell line were isolated and solubilized with RIPA lysis buffer (Sigma-Aldrich, St. Louis, MO, USA) containing a protease inhibitor cocktail (cOmpleteTM Mini EDTA-free, Roche, Basel, Switzerland) and phosphatase inhibitors (PhosSTOP EASYpack, Roche, Basel, Switzerland). Cells were incubated on ice for 1 h and centrifuged at 15,000 rpm for 10 min at 4 °C. Protein concentrations were determined using Bradford reagent (BioRad, Berkeley, CA, USA) according to the manufacturer’s instructions. Proteins were separated by 7.5–15% SDS-PAGE according to the molecular weight of the protein of interest, and transferred to a PVDF membrane using a miniProtean electrophoretic system at 400 mA (BioRad, Berkeley, CA, USA) and a wet electroblotting system (BioRad, Berkeley, CA, USA), respectively. The membrane was incubated overnight at 4 °C with primary antibodies against HSP90α (ab79849, Abcam, Cambridge, UK), HSP90β (ab53497, Abcam, Cambridge, UK), HSP70 (ab45133, Abcam, Cambridge, UK), GRP94 (ab18055, Abcam, Cambridge, UK), CDK4 (ab3112, Abcam, Cambridge, UK), HSP27 (#2402, Cell Signaling, Danvers, MA, USA), EGFR (#4267, Cell Signaling) or ALK (#3633, Cell Signaling). α-Tubulin (T9026, Sigma-Aldrich, St. Louis, MO, USA) or β-actin (A5316, Sigma-Aldrich, St. Louis, MO, USA) was used as a loading control. The membrane was then incubated with horseradish peroxidase-conjugated anti-mouse secondary antibody (#7076, Cell Signaling) or anti-rabbit secondary antibody (#7074, Cell Signaling) for 1 h at room temperature. Protein expression was visualized with an ECL detection reagent (Clarity Western ECL Blotting Substrates (BioRad)) and imaged using chemiluminescence through the ChemiDoc system (BioRad). Densitometric analysis of bands was performed using ImageLab software (BioRad). The ratios between the signals from proteins of in-terest and the loading control were calculated to determine the relative protein expression values. No grouping of gels/blots cropped from different parts of the same gel or from different gels, fields, or exposures was performed.

### 4.5. Sample Preparation for Proteomics Analysis

Cells were seeded at an appropriate density into 10 cm diameter culture dishes to reach 60–70% confluence at 24 h. For each cell line, the proteomic profile of six conditions was identified: 17-AAG, IPI-504, STA-9090, and AUY-922 at IC80 for 24 h, HSP90α + β siRNAs, and untreated cells. Cell lysis and protein extraction were performed as described by Marrugal et al. [40].

Protein concentrations were measured using Qubit fluorometric quantitation (Life Technologies), with each sample aliquoted and stored at 80 °C until required.

### 4.6. iTRAQ Labelling

100 µg of peptide mixture from each sample was reduced with 50 mM tris-(2-carboxyetyl) phosphine (TCEP, AB Sciex) for 1 h at 60 °C with shaking. To block cysteine residues, samples were incubated with 200 mM methylmethanethiosulfate (MMTS, AB Sciex) for 20 min at room temperature. Proteolysis was carried out at 37 °C with trypsin (Promega, Fitchburg, WI, USA) in a ratio by weight of 10:1 (enzyme to substrate) in a water bath overnight. Finally, peptides were dried in a Speed Vac concentrator.

8-plex iTRAQ labeling (AB Sciex) was performed according to the manufacturer’s instructions. Briefly, each protein digestion was reconstituted in 1 M TEAB (triethylamonium bicarbonat) and subsequently labeled at room temperature for 2 h with an isobaric amine-reactive tag as follows: untreated cells, 113; 17-AAG treatment, 114; treatment with IPI-504, 115; STA-9090 treatment, 116; treatment with AUY-922, 117; genetic silencing of HSP90α + β, 121. The samples were then pooled, dried at 45 °C, and incubated at 4 °C overnight.

### 4.7. Nano LC-MS/MS Analysis

Before analysis by mass spectrometry (MS), the iTRAQ-labeled samples were de-salted using Oasis HLB C18 cartridges (Waters) and dried using a Speed Vac concentrator. Next, using a total of 13 increasing concentrations (50, 100, 200, 300, 400, 500, 600, 700, 800, and 900 mM and 1, 1.5, and 2 M) of ammonium formate and MCX Oasis columns (Waters), the peptides were prefractionated.

Nanoliquid chromatography (nano LC 1000, Thermo Scientific) was used to sepa-rate the peptides contained in each fraction, while the analysis was carried out by a nanoelectrospray ionization system (Proxeon Biosystems) connected to a Q Ex-active Plus Orbitrap mass spectrometer (Thermo Scientific). For each sample, 13 µL of each of the 13 fractions was loaded, pre-concentrated, and washed in an Acclaim PepMap precolumn (75 µm × 2 cm, nanoViper, C18, 3 µm, 100 Å; Thermo Scientific). Next, an analytical column (75 µm × 15 cm, nanoViper, C18, 2 µm, 100 Å (Acclaim PepMap RSLC; Thermo Scientific)) was used for 240 min at 200 nL/min to separate the peptides.

Immediately after this step, a gradient of buffer A (0.1% formic acid, 100% H_2_O) to buffer B (0.1% formic acid, 100% acetronitrile) was used to elute the peptides. The elution gradient included 0 to 35% of buffer B for 0–220 min, followed by a ramp of 35 to 45% of buffer B for 220–230 min, and then 45 to 95% of buffer B for 230–240 min. MS/MS analysis was performed using the Q Exactive system in the positive-ion and information-dependent acquisition modes. The scanned mass range was 200–1800 *m*/*z*, at a resolution of 70,000 (full width at half maximum at 100 *m*/*z*). Up to 15 precursors with a charge state greater than or equal to two were selected and incorporated into the list of exclusions for 60 s. The higher-energy collisional dissociation (HCD) spectrum was considered for peptide identification and quantification. Finally, to maximize the abundance of iTRAQ reporter ions, HCD fragmentation was carried out with a collision energy of 32%.

### 4.8. MS Data Analysis

Peptides were identified from MS/MS spectra using the Sequest HT search engine and Percolator, embedded into Proteome Discoverer 1.4 software (Thermo Fisher Scientific) and confronted with footprint patterns of the UniProt database for Homo sapiens. The following search parameters were applied: digestion with trypsin; iTRAQ 8-plex peptide label (N-terminal) and iTRAQ 8-plex peptide label (lysine) as fixed modifications; oxidation of methionine and carboxiamidomethylation of cysteine as variable modifications. Afterward, MS/MS scans of iTRAQ-labeled peptides were used to determine the relative abundances of peptides. The mass spectrometry proteomics data have been deposited to the ProteomeXchange Consortium via the PRIDE [77] partner repository with the dataset identifier PXD040170. The ratios of the iTRAQ reporter ion-peak areas reflected the relative abundances of peptides and ultimately proteins in the samples. To be considered quantifiable, proteins had to present at least two unique peptides with a significance score greater than or equal to 95%, a ratio with a *p*-value < 0.05, and a false discovery rate (FDR) less than 2. Finally, for each identified protein, the expression ratios between the different treatments studied and the corresponding untreated control were calculated. These data were then transformed [log2 (x)] and filtered, including only proteins in which at least one study group contained more than 50% valid values.

### 4.9. Bioinformatics Analysis

The bioinformatics tool used to analyze the proteomic data was PERSEUS Software (www.perseus-framework.org). This software allowed exploring, visualizing, and representing the data as well as analyzing them statistically. Concretely, the one-sample *t*-test tool was used to identify differentially expressed proteins in the different study groups; the obtained *p*-values were corrected by Benjamini-Hochberg FDR and represented in a volcano plot. Adjusted *p*-values less than 0.05 were considered statistically significant.

Once the proteins of interest for each study condition were identified, these were grouped into Venn-Euler diagrams using the jvenn program (http://jvenn.toulouse.inra.fr/app/index.html, accessed on 1 July 2022). Next, the PANTHER (Protein ANalysis Through Evolutionary Relationships) database (http://pantherdb.org/) was used to functionally analyze and categorize these proteins according to their biological processes and molecular functions. Also, protein-protein interaction networks were built based on the publicly available Search Tool for the Retrieval of Interacting Genes/Proteins (STRING) database (https://string-db.org/). Only those results with a Benjamini-Hochberg adjusted *p*-value of less than 0.05 were considered statistically significant. Finally, the Kaplan-Meier Porter website (https://kmplot.com), an open-access resource for the analysis of progression and survival, was used to validate the most important results [78]. Specifically, analyses were used to identify differences in expression levels of proteins of interest in lung adenocarcinoma.

## Figures and Tables

**Table 1:** IC80 values of the HSP90 inhibitors used on lung adenocarcinoma cell lines.

Cell Line	HSP90 Inhibitors						
	IC80 17-AAG nM	IC80 IPI-504 nM	IC80 STA-9090 nM	IC80 AUY-922 nM	EGFR	ALK	KRAS
HCC827	105.02	68.58	20.55	16.67	M	WT	WT
H1975	5.03	51.00	18.96	10.38	M	WT	WT
H1650	26.22	15.06	22.64	5.89	M	WT	WT
H3122	104.66	113.45	31.96	36.44	WT	T	WT
H2228	43.55	185.36	16.52	17.95	WT	T	WT
H2009	172.79	135.33	18.63	9.91	WT	WT	M
H358	52.26	18.65	30.96	32.42	WT	WT	M
A549	65.18	77.97	25.24	122.93	WT	WT	M
H1437	14.83	13.89	27.18	11.26	WT	WT	WT
CALU-3	350.93	173.18	73.78	6963.64	WT	WT	WT
H1781	49.38	123.9	39.82	95.15	WT	WT	WT

nM: nanoMolar; M: mutated gene; T: translocated gene; WT: wild type gene.

**Table 2:** Number of proteins with significant changes after treatment with inhibitors or gene silencing of HSP90 (17-AAG, IPI-504, STA-9090, AUY-922) or gene silencing of HSP90 identified in the panel of lung adenocarcinoma cell lines.

Treatment		Expression Change	HCC827	H1975	H1650	H3122	H2228	H2009	H358	A549	H1437	CALU3	H1781
			EGFR mut			EML4-ALK		KRAS mut			TN		
HSP90 inhibition	17-AAG	+	96	133	29	142	122	126	69	165	31	172	89
		−	238	170	109	389	385	269	337	202	28	438	445
	IPI-504	+	107	169	13	168	181	106	103	194	13	195	120
		−	154	301	40	287	370	258	227	204	16	391	473
	STA-9090	+	94	143	7	167	135	78	188	70	22	288	58
		−	164	230	65	119	147	177	149	113	23	276	244
	AUY-922	+	140	128	31	269	142	79	116	189	56	197	138
		−	167	159	118	202	199	146	161	182	23	483	318
Genetic silencing	siRNA HSP90α + β	+	471	134	69	321	304	269	249	219	92	316	198
		−	243	272	69	309	434	108	289	252	217	270	294

mut: mutated; TN: EGFR, ALK and KRAS wild type; siRNA: small interfering RNA.

**Table 3:** Functional annotation of HSP90 inhibition in different molecular subtypes of lung adenocarcinoma.

Molecular Background	Significant Proteins	Final Proteins *	Biological Pathways
EGFR MUT	332	246	7
EML4-ALK	57	34	10
KRAS MUT	1696	1157	21
TN	1288	866	12

* Significant proteins matched after gene silencing; mut: mutated; TN: EGFR, ALK and KRAS wild type.

**Table 4:** Biological pathways related to differentially expressed proteins following HSP90 inhibition.

EGFR Mut	EML4-ALK	KRAS Mut	Triple Negative
Apoptosis	Apoptosis	Apoptosis	Apoptosis
Arginine biosynthesis	Arginine biosynthesis	Cholesterol biosynthesis	Coenzyme A biosynthesis
Asparagine and aspartate biosynthesis	Formyltetrahydroformate biosynthesis	Purine biosynthesis	Proline biosynthesis
Serine glycine biosynthesis	Succinate to propionate conversion	Pyrimidine ribonucleotides byosinthesis	Huntington’s disease
Methylcitrate cycle	EGFR signaling pathway	Heme biosynthesis	Pyrimidine metabolism
Huntington’s disease	FGF signaling pathway	Insulin/IGF-PKB pathway	DNA replication
p53 pathway	PDGF signaling pathway	Cell cycle	Ubiquitin proteasome pathway
	AR-α signaling pathway	Huntington’s disease	Pentose phosphate pathway
	GnRHR pathway	Parkinson’s disease	L signaling pathway
	Methylmalonil pathway	Axon guidance mediated by semaphorins	PDGF signaling pathway
		CCKR signaling map	Endothelin signaling pathway
		Salvage pyrimidine ribonucleotides	GnRHR pathway
		Cytoskeletal regulation by Rho GTPase	
		DNA replication	
		Ubiquitin proteasome pathway	
		p53 pathway by glucose deprivation	
		EGFR signaling pathway	
		FGF signaling pathway	
		VEGF signaling pathway	
		Cadherin signaling pathway	
		Integrin signaling pathway	

Mut: mutated; Triple Negative: EGFR, ALK and KRAS wild type; EGFR: epidermal growth factor receptor; FGF: fibroblast growth factor; PDGF: platelet-derived growth factor; AR-α: alpha-adrenergic receptor; GnRHR: Gonadotropin-releasing hormone receptor; CCKR: cholecystokinin receptor; VEGF: vascular endothelial growth factor; IL: interleukins.

## Data Availability

Data are available via ProteomeXchange with identifier PXD040170.

## Supplementary Materials

The following supporting information can be downloaded at: https://www.mdpi.com/article/10.3390/ijms241813830/s1.
